# Detection of Colistin Sulfate on Piglet Gastrointestinal Tract Microbiome Alterations

**DOI:** 10.3390/vetsci9120666

**Published:** 2022-11-29

**Authors:** Shulin Fu, Yuzhen Yuan, Xinyue Tian, Linglu Zhou, Ling Guo, Dan Zhang, Jing He, Chun Peng, Yinsheng Qiu, Chun Ye, Yu Liu, Bingbing Zong

**Affiliations:** 1 Hubei Key Laboratory of Animal Nutrition and Feed Science, Wuhan Polytechnic University, Wuhan 430023, China; 2 Hubei Collaborative Innovation Center for Animal Nutrition and Feed Safety, Wuhan 430023, China

**Keywords:** colistin sulfate, 16s rDNA-based metagenome analyses, piglets, gut, microbiome

## Abstract

**Simple Summary:**

The gut microbiome plays important roles in maintaining host health. Overuse of antibiotics could cause microbial resistance and host intestinal flora dysfunction, contributing to serious disease. Colistin is widely used as a feed additive to enhance swine growth and the phenomenon of colistin resistance has been widely reported. Therefore, in this study we wanted to evaluate the effect of colistin on the gastrointestinal tract microbiome composition in piglets. Our results demonstrated that colistin could change the gastrointestinal tract microbiome composition of piglets, which provides us with a new strategy for rational utilization of colistin to protect animal and human health.

**Abstract:**

The gut microbiome exerts important functions on host health maintenance, whereas excessive antibiotic use may cause gut flora dysfunction resulting in serious disease and dysbiosis. Colistin is a broad-spectrum antibiotic with serious resistance phenomena. However, it is unclear whether colistin alters the gastrointestinal tract microbiome in piglets. In this study, 16s rDNA-based metagenome analyses were used to assess the effects of colistin on the modification of the piglet microbiome in the stomach, duodenum, jejunum, cecum, and feces. Both α- and β-diversity indices showed that colistin modified microbiome composition in these gastrointestinal areas. In addition, colistin influenced microbiome composition at the phylum and genus levels. At the species level, colistin upregulated *Mycoplasma hyorhinis*, *Chlamydia trachomatis*, *Lactobacillus agilis*, *Weissella paramesenteroides*, and *Lactobacillus salivarius* abundance, but downregulated *Actinobacillus indolicus*, *Campylobacter fetus*, *Glaesserella parasuis*, *Moraxella pluranimalium*, *Veillonella caviae*, *Neisseria dentiae*, and *Prevotella disiens* abundance in stomachs. Colistin-fed piglets showed an increased abundance of *Lactobacillus mucosae*, *Megasphaera elsdenii DSM 20460*, *Fibrobacter intestinalis*, and *Unidentified rumen bacterium 12-7*, but *Megamonas funiformis*, *Uncultured Enterobacteriaceae bacterium*, *Actinobacillus porcinus*, *Uncultured Bacteroidales bacterium*, and *Uncultured Clostridiaceae bacterium* abundance was decreased in the cecum. In feces, colistin promoted *Mucispirillum schaedleri*, *Treponema berlinense*, *Veillonella magna*, *Veillonella caviae*, and *Actinobacillus porcinus* abundance when compared with controls. Taken together, colistin modified the microbiome composition of gastrointestinal areas in piglets. This study provides new clinical rationalization strategies for colistin on the maintenance of animal gut balance and human public health.

## 1. Introduction

Colistin is an antibiotic with a narrow bactericidal activity spectrum against the most common Gram-negative Enterobacteriaceae and has specific antibacterial activity against members of the Enterobacteriaceae family [1].

Colistin was also the last drug used to treat critical human infections caused by multidrug-resistant Gram-negative bacteria [2]. With antibiotic overuse in recent years, colistin resistance has been extensively identified in the environment, animals, and humans [3]. Antibiotic resistance is a global public health concern as it may spread by horizontal transmission via food chains. Recent studies showed that the combined carriage of *mcr-1* and *mcr-2* genes was identified in the urinary tract pathogens, *Escherichia coli* and *Klebsiella pneumoniae* [4]. In Iran, the *mcr-3* to *mcr-9* genes conferred colistin resistance on pathogenic *E. coli* strains isolated from food-producing animals [2]. Thus, due to an increasing risk of antibiotic resistance in humans and animals, a rational use for colistin must be determined.

Colistin is widely used as a feed additive during swine and poultry production to promote animal growth [5]. Dietary colistin decreases interleukin-1β and tumor necrosis factor-α secretion in weaned piglets [6]. However, recent studies showed that colistin altered the distal gut microbiota of weaned piglets to influence productive and health parameters [7,8]. Colistin also altered the human intestinal microbiota and antibiotic resistome in a simulated human intestinal microbiota model [9] and affected the fecal microbiome composition of broilers [10]. As the microbiome has key roles maintaining host gut immunity, metabolism, and health [11,12], dysfunctional and/or imbalanced gastrointestinal tract microbiota could cause serious disease, including ulcerative colitis [13]. However, it is unclear whether colistin alters the gastrointestinal tract microbiome in piglets.

We investigated the efficacy of colistin on gastrointestinal tract composition of microbiome alterations in piglets, and demonstrated that colistin modified stomach, duodenum, jejunum, cecum, and fecal microbiome composition, and possibly identified important strategies for clinical and rational drug use in animals.

## 2. Materials and Methods

### 2.1. Piglets and Drugs

This experiment was instigated in strict accordance with the recommendations of the China Regulations for the Administration of Affairs Concerning Experimental Animals 1988 and the Hubei Regulations for the Administration of Affairs Concerning Experimental Animals 2005. The protocol was authorized by Hubei Province Science and Technology Department of China (permit number SYXK [ER] 2010-0029). Animal studies were ratified by the Animal Care and Use Committee of Wuhan Polytechnic University, Hubei Province, China (EM952, 6th November 2020). All experimental animals were euthanized in the final experiment.

Ten 28-day-old naturally farrowed early-weaned piglets (Duroc × Landrace × large white), weighing 8–10 kg, were bought from Wuhan Wannianqing Animal Husbandry Co., Ltd. (Wuhan, China) for in vivo studies. Colistin sulfate was purchased from Livzon Group Fuzhou Fuxing Pharmaceutical Co., Ltd. (Fuzhou, China).

### 2.2. Study Design

Piglets were randomly divided into two groups and each group had five. One group was given feed supplemented with 20 g/t colistin sulfate [14], and designated the colistin group. The control group was administrated common animal feed not containing colistin sulfate. After rearing for 14 days, stomach, jejunum, duodenum, cecum, and feces samples not from digesta or tissue, were collected for 16s rDNA sequencing.

### 2.3. DNA Isolation

Approximately 200 mg of frozen sample was resuspended in 250 µL guanidine thiocyanate, 0.1 M Tris (pH 7.5), and 40 µL 10% N-lauroyl sarcosine. DNA was extracted using a QIAamp DNA Stool Mini Kit (Qiagen, Germantown, MD, USA), according to the manufacturer’s instructions. The concentration and molecular weight of DNA was determined using a Nanodrop (Thermo Scientific, Waltham, MA, USA) and agarose gel electrophoresis, respectively.

### 2.4. Construction of a DNA Sequencing Library and Illumina MiSeq Sequencing

The V3–V4 region of bacterial 16s rDNA was PCR amplified using the following primers: 338F 5′-ACTCCTACGGGAGGCAGCAG-3′ and 806R 5′-GGACTACHVGGGTWTCTAAT-3′. The reaction conditions were: 95 °C for 2 min, followed by 25 cycles at 95 °C for 30 s, 55 °C for 30 s, 72 °C for 30 s, and a final extension at 72 °C for 5 min. PCR reactions were carried out in triplicate: 20 μL final volume containing 4 μL 5 × FastPfu Buffer, 2 μL 2.5 mM dNTPs, 0.8 μL each primer (5 μM), 0.4 μL FastPfu polymerase, and 10 ng template DNA. Amplicons were isolated from 2% agarose gels and purified using an AxyPrep DNA Gel extraction kit (Axygen Biosciences, Union City, CA, USA) according to the manufacturer’s instructions. Amplicon quantity and quality were determined using QuantiFluor™-ST instrumentation (Promega, Madison, WI, USA). Purified amplicons were pooled in equimolar concentrations and pair-end sequenced at 2 × 250 under an Illumina MiSeq platform of 16s rDNA-based metagenome analyses, according to standard instructions.

### 2.5. Processing and Analyzing Sequencing Data

Raw fastq files were demultiplexed and quality-filtered using QIIME (version 1.9.1) software [15]. Operational taxonomic units (OTUs) were clustered using a 97% similarity cutoff in UPARSE software (version 7.1) [16]. Chimeric sequences were verified and discarded by UCHIME [17]. The taxonomies of 16s rDNA gene sequences were determined by RDP Classifier. MOTHUR software and QIIME were used to evaluate diversity within communities (α-diversity) and between community diversity (β-diversity). Sample clustering was performed using the QIIME pipeline (v1.8.0) and the unweighted pairwise grouping method [18]. Heatmaps at different levels were performed using the heatmap tool in R. Venn diagrams were drafted using the VennDiagram tool in R. Principal component analysis (PCA) was conducted using the ade4 tool in R. Unweighted PCoA was performed using QIIM E2. NMDS was calculated using the phyloseq tool in R.

### 2.6. Statistical Analysis

Study results were expressed as the mean ± standard deviation. Differences between groups were determined using one-way ANOVA and *p* values < 0.05 were considered significantly different.

## 3. Results

### 3.1. Detection of Colistin on Piglet Gastrointestinal Tract Composition

**Figure 1:**
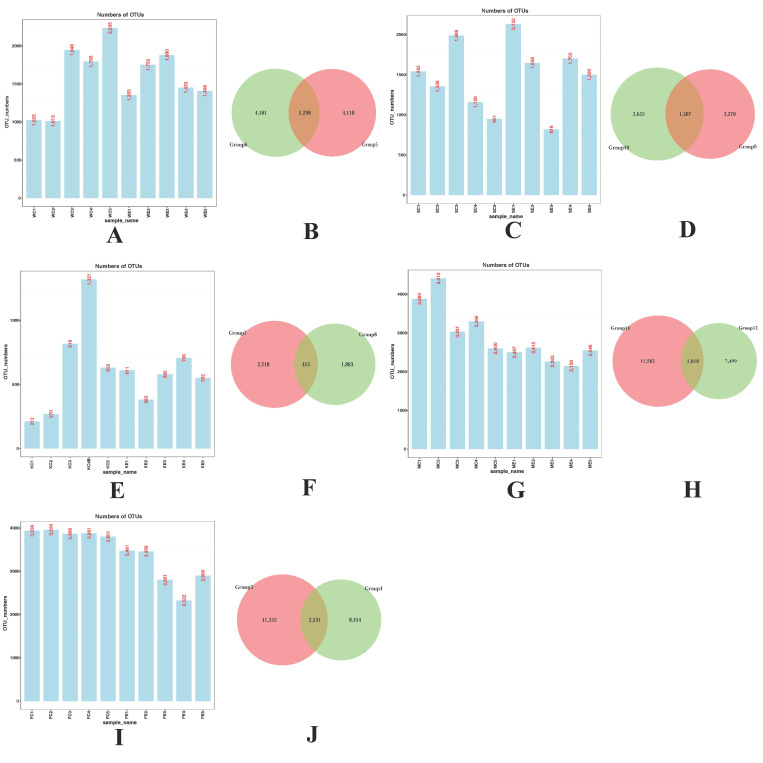
Diagrammatic representation of the effects of colistin on OTU changes in piglet guts (**A**,**C**,**E**,**G**,**I**). Venn diagrams show unique OTUs in the colistin and control groups, and shared by the groups (circle intersection) (**B**,**D**,**F**,**H**,**J**). (**A**) Stomach (Group 6, Group 5); (**B**) duodenum (Group 10, Group 9); (**C**) jejunum (Group 8, Group 7); (**D**) cecum (Group 12, Group 11); (**E**) feces (Group 4, Group 3); colistin treated group: Group 6, group 10, group 8, group 12, and group 4; the control group: Group 5, group 9, group 7, group 11, and group 3.

We used 16s rDNA method to determine the efficacy of colistin on piglet microbiome modifications in the stomach, duodenum, jejunum, cecum, and feces and 7846 ± 232, 7802 ± 476, 2831 ± 118, 12065 ± 197, and 14963 ± 487 OTUs were identified in the colistin treated group, respectively, while 8014 ± 555, 6998 ± 396, 3253 ± 451, 17217 ± 712, and 19443 ± 62 OTUs, respectively, were identified in controls (Figure 1A,C,E,G,I). Using Venn diagrams, we also identified 7569 shared OTUs between the groups with respect to stomach, duodenum, jejunum, cecum, and feces (Figure 1B,D,F,H,J).

### 3.2. Alpha Diversity in Piglet Gut Microbiome Composition Is Influenced by Colistin

**Figure 2:**
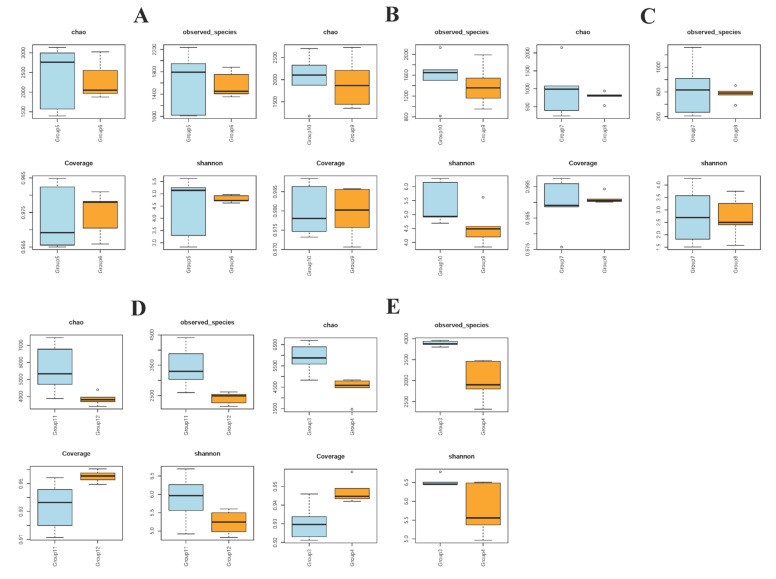
Alpha diversity indices on the effects of colistin on gastrointestinal tract microbiome change in piglets. (**A**) Stomach (Group 6, Group 5); (**B**) duodenum (Group 10, Group 9); (**C**) jejunum (Group 8, Group 7); (**D**) cecum (Group 12, Group 11); (**E**) feces (Group 4, Group 3); colistin treated group: Group 6, group 10, group 8, group 12, and group 4; control group: Group 5, group 9, group 7, group 11, and group 3.

For α-diversity, four different analysis methods were used: chao, observed species, coverage, and Shannon indices. When compared with the control group, all indices in the duodenum, cecum, and feces were increased in the colistin group (*p* < 0.05, Figure 2B,D,E). However, all indices in the colistin group for stomach and jejunum, and chao, observed species, and Shannon indices from cecum and feces were decreased (*p* < 0.05, Figure 2A,C–E).

### 3.3. Beta Diversity in Piglet Gut Microbiome Composition Is Influenced by Colistin

**Figure 3:**
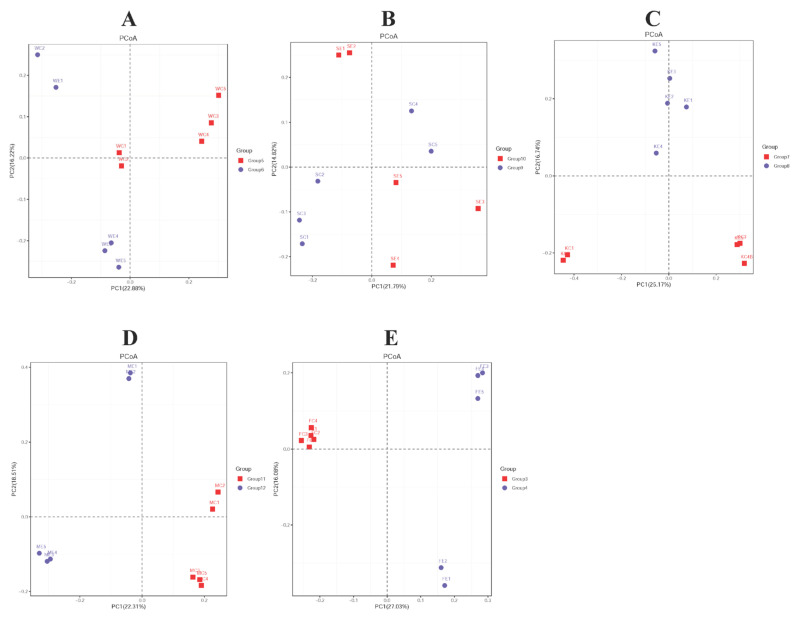
PCoA analysis of the efficacy of colistin on piglet gastrointestinal tract microbiome change. (**A**) Stomach (Group 6, Group 5); (**B**) duodenum (Group 10, Group 9); (**C**) jejunum (Group 8, Group 7); (**D**) cecum (Group 12, Group 11); (**E**) feces (Group 4, Group 3); colistin treated group: Group 6, group 10, group 8, group 12, and group 4; control group: Group 5, group 9, group 7, group 11, group 3. Group 3 (FC1, FC2, FC3, FC4, FC5), Group 4 (FE1, FE2, FE3, FE4, FE5), Group 5 (WC1, WC2, WC3, WC4, WC5), Group 6 (WE1, WE2, WE3, WE4, WE5), Group 7 (KC1, KC2, KC3, KC4, KC5), Group 8 (KE1, KE2, KE3, KE4, KE5), Group 9 (SC1, SC2, SC3, SC4, SC5), Group10 (SE1, SE2, SE3, SE4, SE5), Group 11 (MC1, MC2, MC3, MC4, MC5), Group 12 (ME1, ME2, ME3, ME4, ME5).

In terms of β-diversity, PCOA, PCA, and NMDS analyses were used to determine the effects of colistin on the piglet gut microbiome. For PCoA and PCA analyses, most stomach, duodenum, jejunum, cecum, and feces samples from both groups were clustered together (Figure 3A,C–E, Supplementary Figure S1A,B,D,E). However, some samples were separated (Figure 3B, Supplementary Figure S1C). NMDS-based analyses, based on Bray-Curtis distances, showed that gut microbiome composition was highly diverse when piglets were treated with colistin, and displayed location-specific manners (Supplementary Figure S2A,B,D,E) except for jejunum samples (Supplementary Figure S2C).

### 3.4. The Efficacy of Colistin on Piglet Microbiome Change at Phylum Levels

**Figure 4:**
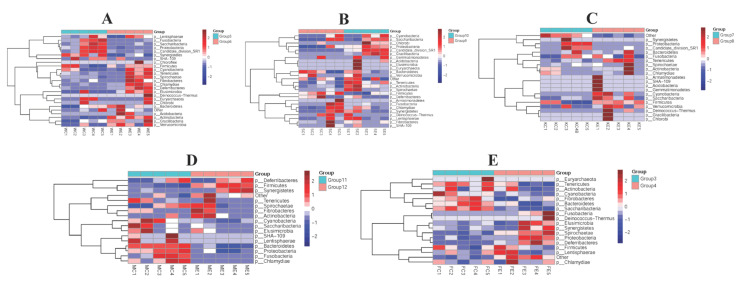
Heatmap showing the efficacy of colistin on piglet microbiome change at phylum levels. (**A**) Stomach (Group 6, Group 5); (**B**) duodenum (Group 10, Group 9); (**C**) jejunum (Group 8, Group 7); (**D**) cecum (Group 12, Group 11); (**E**) feces (Group 4, Group 3); colistin treated group: Group 6 (WE1, WE2, WE3, WE4, WE5), group 10 (SE1, SE2, SE3, SE4, SE5), group 8 (KE1, KE2, KE3, KE4, KE5), Group 12 (ME1, ME2, ME3, ME4, ME5), and Group 4 (FE1, FE2, FE3, FE4, FE5); control group: Group 5 (WC1, WC2, WC3, WC4, WC5), group 9 (SC1, SC2, SC3, SC4, SC5), Group 7 (KC1, KC2, KC3, KC4, KC5), Group 11 (MC1, MC2, MC3, MC4, MC5), and Group 3 (FC1, FC2, FC3, FC4, FC5).

Stomach, duodenum, jejunum, cecum, and feces samples in both groups were collected to assess gut microbiome composition at phylum levels. Colistin changed the overall microbiome composition at the phylum level (Figure 4). Spirochaetae, Chlamydiae, Deferribacteres, Cyanobacteria, and Elusimicrobia abundance in stomach samples from the colistin group were significantly upregulated when compared with controls (Figure 4A). However, the dominant abundant phyla in the control stomach group were Fusobacteria, Saccharibacteria, Proteobacteria, Candidate division SR1, and Synergistetes (Figure 4A).

In the duodenum, colistin increased Candidate division SR1, Gracilibacteria, Tenericutes, and Proteobacteria abundance when compared with controls (Figure 4B). In control jejunum, the main abundant phyla were Proteobacteria and Candidate division SR1, while Tenericutes, Cyanobacteria, Saccharibacteria, and Firmicutes were the main phyla in the colistin group (Figure 4C).

### 3.5. The Efficacy of Colistin on Piglet Microbiome Change at Genus Levels

**Figure 5:**
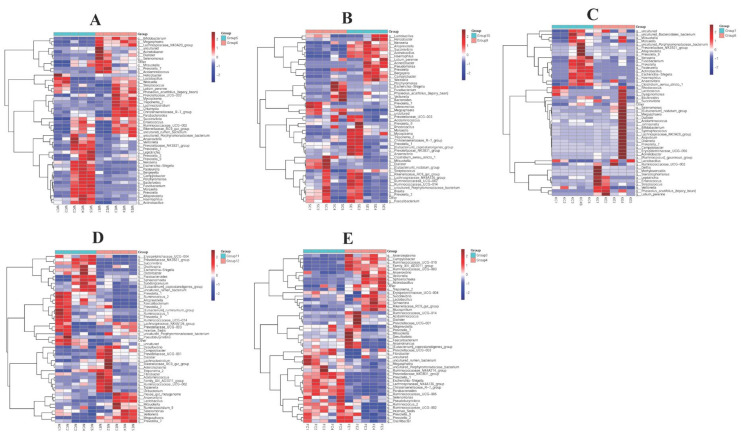
Heatmap showing the efficacy of colistin on piglet microbiome change at genus levels. (**A**) Stomach (Group 6, Group 5); (**B**) duodenum (Group 10, Group 9); (**C**) jejunum (Group 8, Group 7); (**D**) cecum (Group 12, Group 11); (**E**) feces (Group 4, Group 3); colistin treated group: Group 6 (WE1, WE2, WE3, WE4, WE5), group 10 (SE1, SE2, SE3, SE4, SE5), group 8 (KE1, KE2, KE3, KE4, KE5), Group 12 (ME1, ME2, ME3, ME4, ME5), and Group 4 (FE1, FE2, FE3, FE4, FE5); control group: Group 5 (WC1, WC2, WC3, WC4, WC5), Group 9 (SC1, SC2, SC3, SC4, SC5), Group 7 (KC1, KC2, KC3, KC4, KC5), Group 11 (MC1, MC2, MC3, MC4, MC5), and Group 3 (FC1, FC2, FC3, FC4, FC5).

We also determined the effects of colistin on piglet microbiome changes at genus levels. When piglets were fed colistin, Bifidobacterium, Megasphaera, Lachnospiraceae NK3A20 group, Treponema 2, Lachnoclostridium, and Parabacteroides were dominantly abundant in the stomach, but in controls Escherichia-Shigella, Pasteurella, Bergeyella, Haemophilus, and Actinobacillus abundance was significantly upregulated (Figure 5A).

In the cecum, colistin increased Anaerovibrio, Lactobacillus, Selenomonas, Veillonella, Megasphaera, and Prevotella 7 abundance when compared with controls (Figure 5C). However, in feces, Ruminococcaceae NK4A214 group, Prevotellaceae NK3B31 group, Prevotella 1, Escherichia-Shigella, Lachnospiraceae NK4A136 group, Christensenellaceae R-7 group, Parabacteroides, and Ruminococcaceae UCG-005 abundance was significantly decreased in the colistin group, but Campylobacter, Ruminococcaceae UCG-010, Family XIII AD3011 group, Ruminococcaceae UCG-003, and Anaerovibrio abundance was upregulated when compared with controls (*p* < 0.05, Figure 5E).

### 3.6. The Efficacy of Colistin on Piglet Microbiome Change at Species Levels

**Figure 6:**
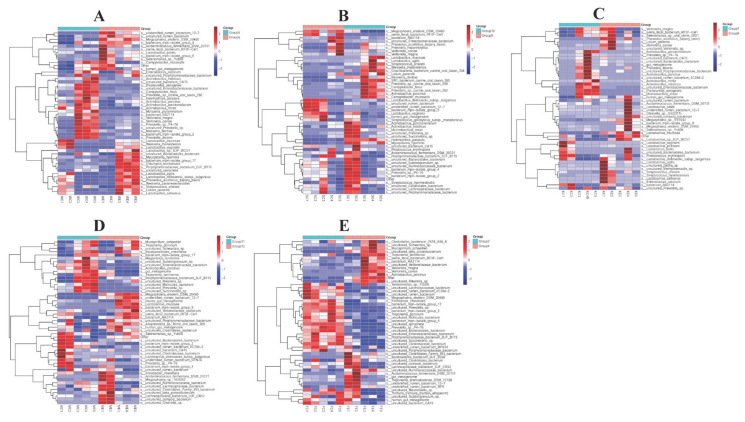
Heatmap showing the efficacy of colistin on piglet microbiome change at species levels. (**A**) Stomach (Group 6, Group 5); (**B**) duodenum (Group 10, Group 9); (**C**) jejunum (Group 8, Group 7); (**D**) cecum (Group 12, Group 11); (**E**) feces (Group 4, Group 3); the colistin treated group: Group 6 (WE1, WE2, WE3, WE4, WE5), Group 10 (SE1, SE2, SE3, SE4, SE5), Group 8 (KE1, KE2, KE3, KE4, KE5), Group 12 (ME1, ME2, ME3, ME4, ME5), and Group 4 (FE1, FE2, FE3, FE4, FE5); the control group: Group 5 (WC1, WC2, WC3, WC4, WC5), Group 9 (SC1, SC2, SC3, SC4, SC5), Group 7 (KC1, KC2, KC3, KC4, KC5), Group 11 (MC1, MC2, MC3, MC4, MC5), and Group 3 (FC1, FC2, FC3, FC4, FC5).

Samples were analyzed to explore piglet gut microbiome changes at species levels. In the stomach, colistin promoted *Mycoplasma hyorhinis*, *Chlamydia trachomatis*, *Lactobacillus agilis*, *Weissella paramesenteroides*, and *Lactobacillus salivarius* abundance, and reduced *Actinobacillus indolicus*, *Campylobacter fetus*, *Glaesserella parasuis* (*G. parasuis*, *Haemophilus parasuis*), *Moraxella pluranimalium*, *Veillonella caviae*, *Neisseria dentiae*, and *Prevotella disiens* levels (Figure 6A). However, the abundance of *Lactobacillus mucosae*, *Megasphaera elsdenii DSM 20460*, *Fibrobacter intestinalis*, and *Unidentified rumen bacterium 12-7* was upregulated, but *Megamonas funiformis*, *Uncultured Enterobacteriaceae bacterium*, *Actinobacillus porcinus, Uncultured Bacteroidales bacterium*, and *Uncultured Clostridiaceae* abundance in the cecum in the colistin group was downregulated (Figure 6D).

In feces, Mucispirillum schaedleri, Treponema berlinense, Veillonella magna, Veillonella caviae, and Actinobacillus porcinus abundance was increased (Figure 6E), while Megasphaera elsdenii DSM 20460, Fibrobacter intestinalis, Treponema porcinum, uncultured Mollicutes bacterium, and uncultured Enterobacteriaceae abundance was decreased when compared with controls (Figure 6E).

## 4. Discussion

We explored the efficacy of colistin sulfate on piglet gastrointestinal tract microbiome composition. Colistin sulfate is as an alkaline polypeptide antibiotic and interacts with bacterial cell membranes to alter membrane permeability, leading to cell death and antibacterial effects [19,20]. The antibiotic is mainly used to prevent and control bacterial infections and promote livestock growth. Due to its extensive use, drug resistance to colistin has become extremely serious, endangering human and animal health [21]. New World Health Organization guidelines on the use of antibiotics in food animals has helped maintain antibiotic effectiveness by reducing their use in animals to promote healthy growth and prevent disease [22]. Previous research also indicated that colistin was nephrotoxic as it increased membrane permeability and stress oxidation, thereby contributing to apoptosis [23]. It was also shown that colistin-resistant *E. coli* and the *mcr* gene family were transmitted through food [24]. Other reports demonstrated that gut microbiota regulated host nutrition, immunity, and health [25]. Therefore, whether colistin sulfate alters the gastrointestinal tract microbiome, and whether these modifications are associated with health, requires more study.

Our research remit was to investigate the effects of colistin sulfate on the gut microbiota in piglets. The gastrointestinal tract is thought to be the largest immune organ in the body, and plays core roles in balancing immune dynamics [26]. Previous research showed that intestinal microorganisms maintain stability in host intestinal environments [27]. In our study, when piglets were fed colistin sulfate, Proteus and Candidate division SR1 phyla abundance in the stomach decreased, while levels in the duodenum and jejunum increased. Previous research indicated that Proteus proliferation in the intestinal tract reflected unstable structures in intestinal microbial communities [28]. In addition, antibiotic treatment resulted in septic rats, displaying higher abundance of Proteus [29]. Vancomycin could significantly increase the level of Proteus in mice gut [30]. In our study, Proteus abundance exhibited different trends in different intestinal sections. Whether alterations in Proteus abundance induced by colistin sulfate were related to intestinal health requires further investigation.

From sequencing, the gastrointestinal tract microbiome was changed in the colistin group. Mycoplasma abundance in stomach, and Lactobacillus and *Chlamydia trachomatis* abundance in the stomach and cecum was upregulated when piglets were fed colistin.

Previous research reported that Lactobacillus naturally existed in the gastrointestinal tract and oral cavity of humans and animals [31], and exerted good antibacterial effects toward gastrointestinal pathogens and rotavirus [32]. Previous studies also showed that Lactobacillus potentially functioned as a probiotic to regulate host cell immunity and anti-inflammatory responses [33]. *Lactobacillus mucosae* was upregulated in the cecum in the colistin group. *Lactobacillus mucosae* exerted antiviral effects on respiratory syncytial virus infection in mice [34]. Feeding *Lactobacillus mucosae* could enhance feed efficiency and ileal morphological structure during *Escherichia coli* LPS challenge [35]. However, the impact of increased Lactobacillus abundance, as triggered by colistin, on host gut health requires further exploration.

Mycoplasma mainly parasitizes the epithelial surface and serosa of respiratory and genitourinary tracts [36], thus resisting phagocytosis by the host immune system [37]. It was previously shown that Chlamydia was unable to synthesize lipopolysaccharide and folic acid, thereby contributing to resistance to polymyxin and sulfonamides [38]. It remains unclear if *Chlamydia trachomatis* upregulation in the gastrointestinal tract of colistin sulfate treated piglets was associated with drug resistance.

Interestingly and abnormally, *G. parasuis* was identified in the stomach of piglets treated with colistin sulfate. *G. parasuis* is responsible for Glässer’s disease in pigs and dwells in the upper respiratory tract of healthy animals [39]. The bacteria cause severe inflammatory reactions, such as arthritis and meningitis [40]. We hypothesize that this *G. parasuis* presence in piglets fed colistin sulfate may have been due to imbalanced gastrointestinal tract flora and the promotion of abnormal flora growth. Future studies are required to verify this hypothesis.

## 5. Conclusions

Taken together, colistin sulfate altered piglet gut microbiome composition. These data enhance our understanding of colistin sulfate functions in regulating the piglet gut microbiome. Our research also provides new strategies to clinically rationalize colistin sulfate for improving animal gut balance and human public health.

## Data Availability

Not applicable.

## Supplementary Materials

The following supporting information can be downloaded at: https://www.mdpi.com/article/10.3390/vetsci9120666/s1, Figure S1: PCA analysis of the effects of colistin on piglet gut microbiome composition. (A) Stomach (Group 6, Group 5); (B) duodenum (Group 10, Group 9); (C) jejunum (Group 8, Group 7); (D) cecum (Group 12, Group 11); (E) feces (Group 4, Group 3); colistin treated group: Group 6, group 10, group 8, group 12, and group 4; control group: Group 5, group 9, group 7, group 11, and group 3; Figure S2: NMDS analysis of the effects of colistin on piglet gut microbiome composition. (A) Stomach (Group 6, Group 5); (B) duodenum (Group 10, Group 9); (C) jejunum (Group 8, Group 7); (D) cecum (Group 12, Group 11); (E) feces (Group 4, Group 3); colistin treated group: Group 6, group 10, group 8, group 12, and group 4; control group: Group 5, group 9, group 7, group 11, and group 3. Group 3 (FC1, FC2, FC3, FC4, FC5), Group 4 (FE1, FE2, FE3, FE4, FE5), Group 5 (WC1, WC2, WC3, WC4, WC5), Group 6 (WE1, WE2, WE3, WE4, WE5), Group 7 (KC1, KC2, KC3, KC4, KC5), Group 8 (KE1, KE2, KE3, KE4, KE5), Group 9 (SC1, SC2, SC3, SC4, SC5), Group10 (SE1, SE2, SE3, SE4, SE5), Group 11 (MC1, MC2, MC3, MC4, MC5), Group 12 (ME1, ME2, ME3, ME4, ME5).
