# Supplementary material for: Detection of Colistin Sulfate on Piglet Gastrointestinal Tract Microbiome Alterations
Source: Vet Sci. 2022 Nov 29;9(12):666. doi: 10.3390/vetsci9120666 (PMC9787881; doi:10.3390/vetsci9120666)

Figure S1: PCA analysis of the effects of colistin on piglet gut microbiome composition. (A) Stomach (Group 6, Group 5); (B) duodenum (Group 10, Group 9); (C) jejunum (Group 8, Group 7); (D) cecum (Group 12, Group 11); (E) feces (Group 4, Group 3); colistin treated group: Group 6, group 10, group 8, group 12, and group 4; control group: Group 5, group 9, group 7, group 11, and group 3; Group 3 (FC1, FC2, FC3, FC4, FC5), Group 4 (FE1, FE2, FE3, FE4, FE5), Group 5 (WC1, WC2, WC3, WC4, WC5), Group 6 (WE1, WE2, WE3, WE4, WE5), Group 7 (KC1, KC2, KC3, KC4, KC5), Group 8 (KE1, KE2, KE3, KE4, KE5), Group 9 (SC1, SC2, SC3, SC4, SC5), Group10 (SE1, SE2, SE3, SE4, SE5), Group 11 (MC1, MC2, MC3, MC4, MC5), Group 12 (ME1, ME2, ME3, ME4, ME5) .

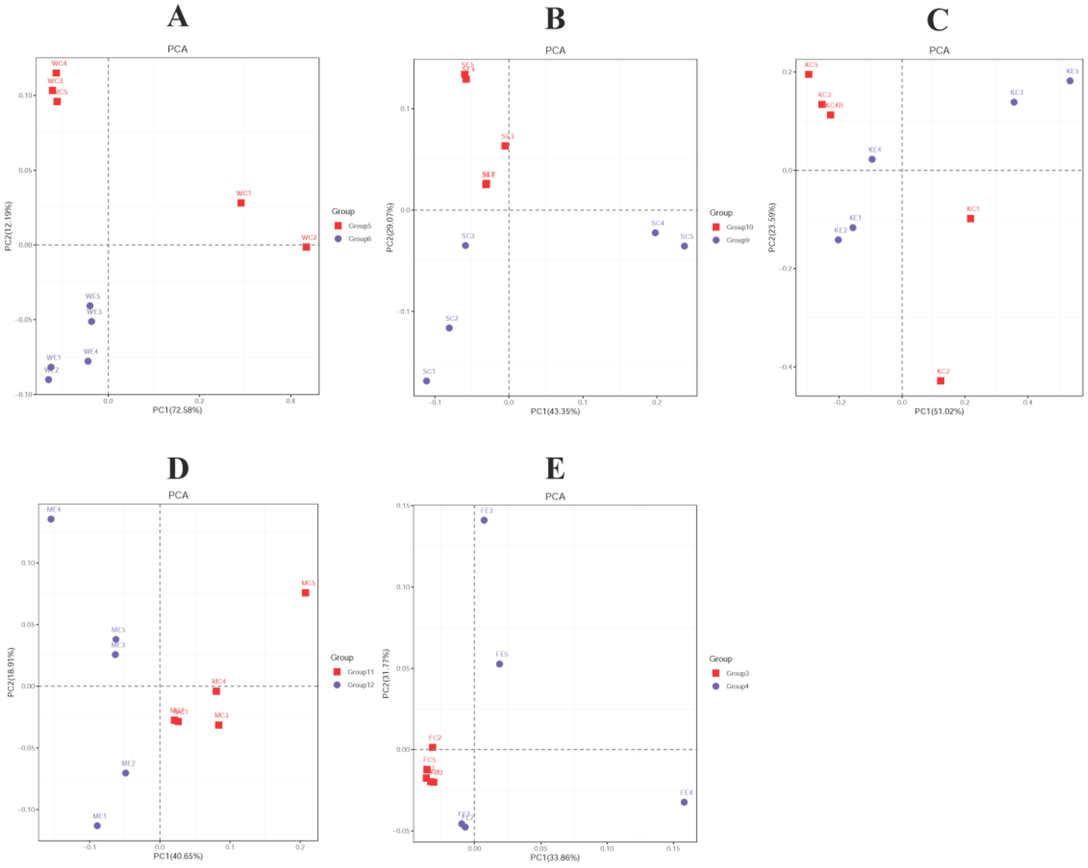

Figure S2: NMDS analysis of the effects of colistin on piglet gut microbiome composition. (A) Stomach (Group 6, Group 5); (B) duodenum (Group 10, Group 9); (C) jejunum (Group 8, Group 7); (D) cecum (Group 12, Group 11); (E) feces (Group 4, Group 3); colistin treated group: Group 6, group 10, group 8, group 12, and group 4; control group: Group 5, group 9, group 7, group 11, and group 3. Group 3 (FC1, FC2, FC3, FC4, FC5), Group 4 (FE1, FE2, FE3, FE4, FE5), Group 5 (WC1, WC2, WC3, WC4, WC5), Group 6 (WE1, WE2, WE3, WE4, WE5), Group 7 (KC1, KC2, KC3, KC4, KC5), Group 8 (KE1, KE2, KE3, KE4, KE5), Group 9 (SC1, SC2, SC3, SC4, SC5), Group10 (SE1, SE2, SE3, SE4, SE5), Group 11 (MC1, MC2, MC3, MC4, MC5), Group 12 (ME1, ME2, ME3, ME4, ME5) .

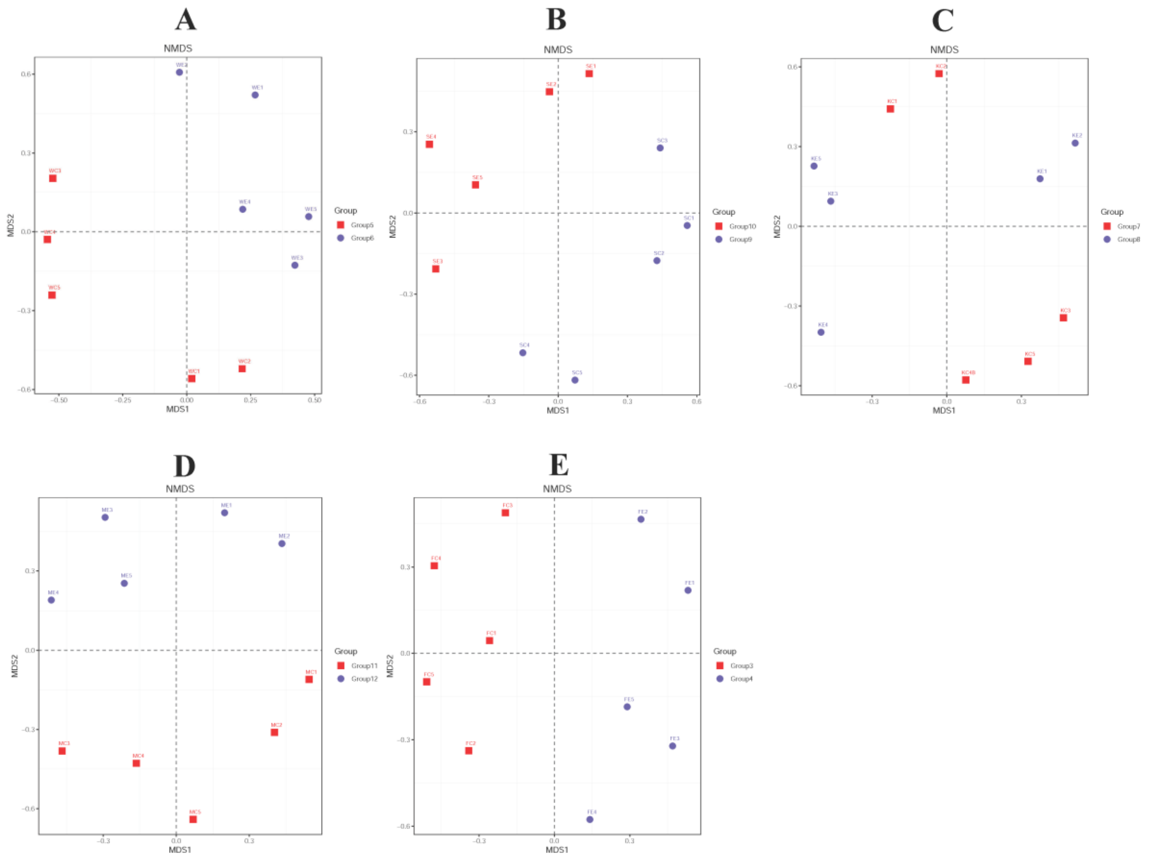

Supplement: Supplementary file 1 [file vetsci-09-00666-s001.zip › vetsci-2004510-supplementary.pdf]
